# Characterization and functional analysis of *SIAH1* during skin and hair follicle development in the angora rabbit (*Oryctolagus cuniculus*)

**DOI:** 10.1186/s41065-020-00126-0

**Published:** 2020-04-06

**Authors:** Tong Zhou, Yang Chen, Bohao Zhao, Shuaishuai Hu, Jiali Li, Ming Liu, Shuang Liang, Zhiyuan Bao, Xinsheng Wu

**Affiliations:** 1 College of Animal Science and Technology, Yangzhou University, Yangzhou, China; 2 Joint International Research Laboratory of Agriculture and Agri-Product Safety, Yangzhou University, Yangzhou, China

**Keywords:** *SIAH1*, Molecular cloning, Expression analysis, Skin and hair follicle development, Angora rabbit

## Abstract

**Background:**

Seven in absentia homolog 1 (*SIAH1*) is an E3 ubiquitin ligase containing a RING-finger domain and a key regulator of normal development. Skin and hair follicle development is a complex and special process of morphogenesis involving multiple signaling pathways. *SIAH1* is enriched in the Wnt signaling pathway and potentially related to hair follicle cycle and skin development. This study aims to provide evidence for the role of *SIAH1* in skin and hair development.

**Results:**

Full-length cloning and analysis of *SIAH1* was conducted to better understand its function. Phylogenetically, the sequence of SIAH1 in the rabbit shares the greatest homology with *Home sapiens*, *Pongo abelii* and *Mus mulatta.* Based on the rabbit hair follicle synchronization model, we found that the expression level of *SIAH1* in the regressive period of the rabbit hair cycle is significantly lower than in the active growth and rest periods. In addition, the mRNA expression levels of skin and hair follicle development-related genes changed significantly when *SIAH1* was overexpressed and silenced. After *SIAH1* overexpression, the expression levels of *WNT2*, *LEF1* and *FGF2* decreased, and those of *SFRP2* and *DKK1* increased (*P* < 0.05). After interference of *SIAH1*, the expression levels of *WNT2*, *LEF1* and *FGF2* increased (*P* < 0.05), and *SFRP2* and *DKK1* decreased.

**Conclusions:**

*SIAH1* can affect skin and hair follicle development and exert an inhibitory effect. These results could provide foundamental insights into the role of *SIAH1* as a target gene in rabbit skin and hair follicle development.

## Background

Skin and hair follicle development is a complex and special morphogenic process in which multiple signaling pathways and epithelial-mesenchymal interactions are involved [1]. Skin and hair follicle development is a cycling and self-renewal process, consisting of three periods: active growth (anagen), regression (catagen) then rest (telogen) [2, 3]. Dermal papilla (DP) cells are located at the base of the hair follicle and secrete a variety of cytokines that regulate adjacent tissues, thereby regulating hair growth and renewal [4, 5]. Dermal hair papilla cells can be used as a cell model to study hair follicle growth and periodic changes. A number of studies have revealed that different genes have either a promoting or inhibitory effect on skin and hair follicle development, including hepatocyte growth factor (*HGF*), heat shock protein 27 (*HSP27*), secreted frizzled-related protein 4 (*SFRP2*) and fibroblast growth factor 5 (*FGF5*) [6–9]. Previously, differentially expressed genes (DEGs) related to hair follicle cycling were identified by Illumina sequencing in skin samples from different stages of skin and hair follicle developmental in Angora rabbits, including *BMP2*, *KRT17*, *HTATIP2* and *SIAH1* [10].

Although multiple genes that are possibly implicated in the skin and hair development have been identified, the specific role of *SIAH1* in this respect remains unclear. *SIAH1* is an E3 ubiquitin ligase containing a RING-finger domain and a key regulator of normal development. *SIAH1* functions by degrading substrate proteins using the polyubiquitin-proteasome pathway [11]. The Wnt signaling pathway plays an important role in the regulation of skin and hair follicle development, by regulating the hair growth cycle and promoting hair follicle differentiation [12]. *SIAH1* affects the activation of the β-catenin-dependent T cell factor/lymphocyte enhancer (*TCF/LEF*) family of transcription factors by modifying the quantity of β-catenin that is deposited in the nucleus, thereby regulating cell proliferation [13–15]. *SIAH1* displays a variety of cell biological functions, participating in cell cycle regulation, and cellular differentiation and apoptosis [16–18]. In our early studies, we found significant differences in *SIAH1* expression in rabbits at different stages of hair follicle cycle, suggesting that the *SIAH1* may be involved in the periodic growth of hair follicles [10].

In this study, the complete coding sequence (CDS) of *SIAH1* of Angora rabbits was amplified. The sequence characteristics and evolutionary relationship of *SIAH1* were predicted using bioinformatics analysis. In addition, mRNA expression levels of this gene from various tissues were examined at different phases of hair growth. Furthermore, interference and overexpression techniques were utilized to promote and inhibit *SIAH1* mRNA expression levels which, with other genes involved in skin and hair follicle development, were examined in rabbit dermal papilla cells. The results provide evidence for the role of *SIAH1* in skin and hair development.

## Results

### cDNA cloning and sequence analysis of *SIAH1*

**Fig. 1:**
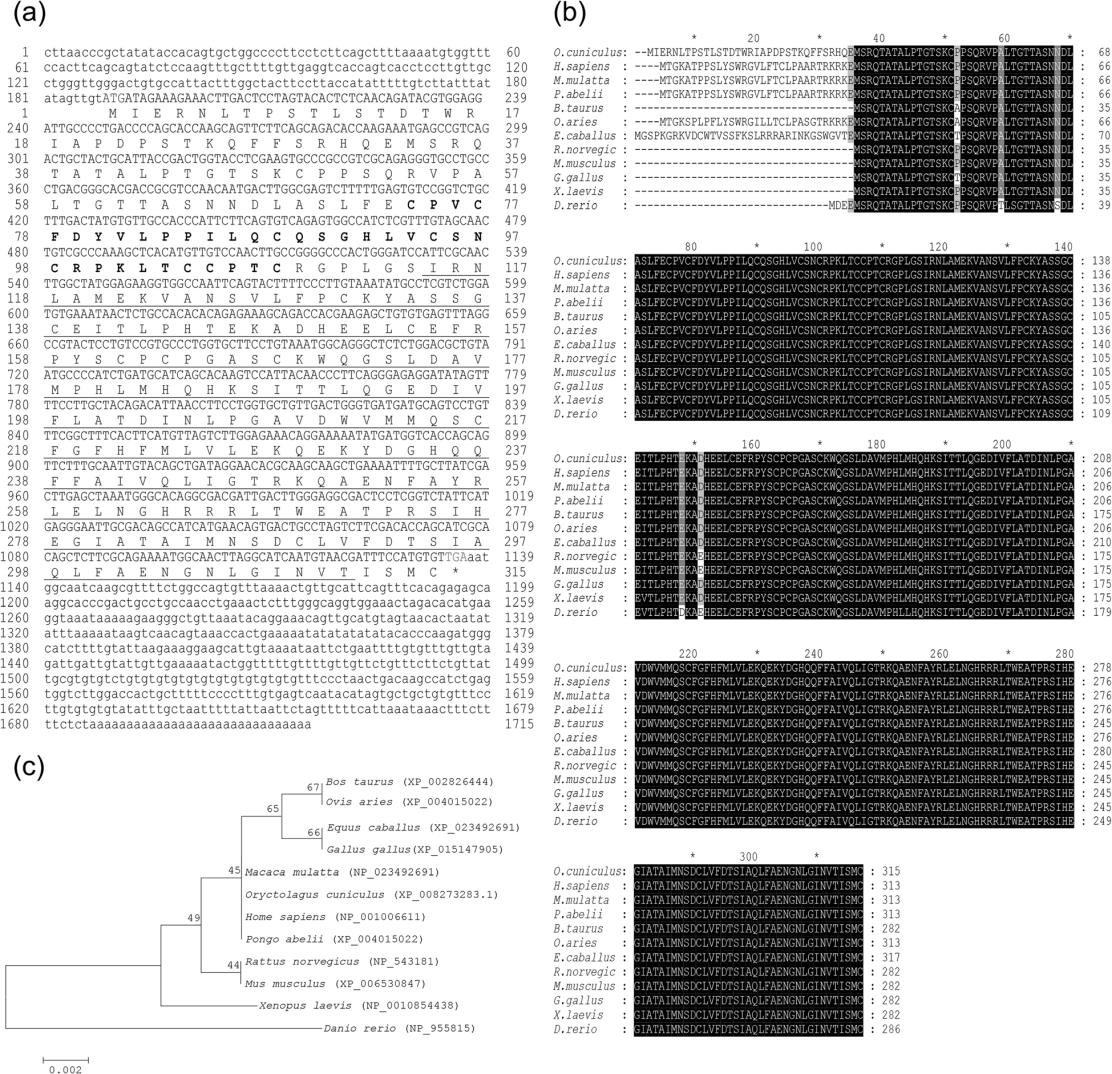
Molecular characterization of *SIAH1*. **a** Nucleotide and deduced amino acid sequences of Angora rabbit *SIAH1* gene. * indicates stop codon; blue letters indicate initiation codon; green letters indicate termination codon; the RING domain is in bold and the Sina domain underlined. **b** Multiple sequence alignment of deduced amino acids of *SIAH1* between rabbit and other species. The black and gray highlights represent identical and similar, respectively. **c** The neighbor-joining phylogenetic tree was constructed based on the *SIAH1* amino acid sequence of each species. The confidence of each node was evaluated repeatedly by 1000 bootstrap with Mega 7.0. The ruler (0.002) means genetic distance

The full length of the cloned *SIAH1* (GenBank accession no. MN520291) was 1715 bp, of which the 5′-untranslated region (UTR) was 188 bp and the 3′-UTR 579 bp. The open reading frame (ORF) had a length of 948 bp and encoded 315 amino acids (Fig. 1a). The analysis indicated that the CDS of *SIAH1* contained RING structural (residues 74–108) and Sina (residues 115–311) domains.

The isoelectric point (PI) and peptide molecular weight predicted by the ProtParam tool were 6.40 and 34.9 kDa. The SIAH1 amino acid sequence had 29 residues with a negative charge and 26 with a positive charge suggesting that the protein as a whole possessed a negative charge. The instability coefficient was estimated to be 46.92, indicating that this protein was classified as an unstable protein. The hydrophobicity analysis indicated that SIAH1 was a hydrophilic protein. According to the results of SignalP 5.0 software analysis, the protein possessed no apparent signaling peptide. TMHMM prediction software demonstrated no significant transmembrane protein domain, indicating that SIAH1 was neither a membrane receptor nor a membrane. SIAH1 was shown to be principally distributed in the nucleus (47.8%) and secondarily within the cytoplasm (34.8%), indicating that SIAH1 performs its biological role mainly in the nucleus. The predicted secondary structure was shown to be 21.59% α-helix, 51.75% random coil, 20.63% extended strand and 6.03% beta turn.

### Multiple sequence alignment and evolutionary relationships of *SIAH1*

The sequence of the rabbit SIAH1 coding region obtained from NCBI was aligned with the multiple amino acid (AA) sequences of SIAH1 from other species using the online tool Blastp. The AA sequences of SIAH1 were 99.65–97.18% identical with *Mus musculus* (XP_006530847), *Rattus norvegicus* (NP_543181), *Bos Taurus* (XP_005218736), *Gallus gallus* (XP_015147905), *Equus caballus* (XP_023492691), *Xenopus laevis* (NP_001085438), *Pongo abelii* (XP_002826444), *Macaca mulatta* (NP_001247767), *Home sapiens* (NP_001006611), *Ovis aries* (XP_004015022) and *Danio rerio* (NP_955815). The two conserved domains of SIAH1 were also found in identical positions, demonstrating that they were highly conserved among different species. The sequence of SIAH1 in rabbits was identical to *Home sapiens*, *Pongo abelii* and *Mus mulatta,* except for the 32 AA N-terminal end of the sequence (Fig. 1b).

NJ-phylogenetic trees were constructed using SIAH1 sequences from rabbits and multiple other species. The amino acid sequence of rabbit SIAH1 possessed the greatest homology with *Home sapiens*, *Pongo abelii* and *Mus mulatta* and the lowest with *Danio rerio* (Fig. 1c).

### Expression profile of *SIAH1* in different tissues and hair follicle cycle

**Fig. 2:**
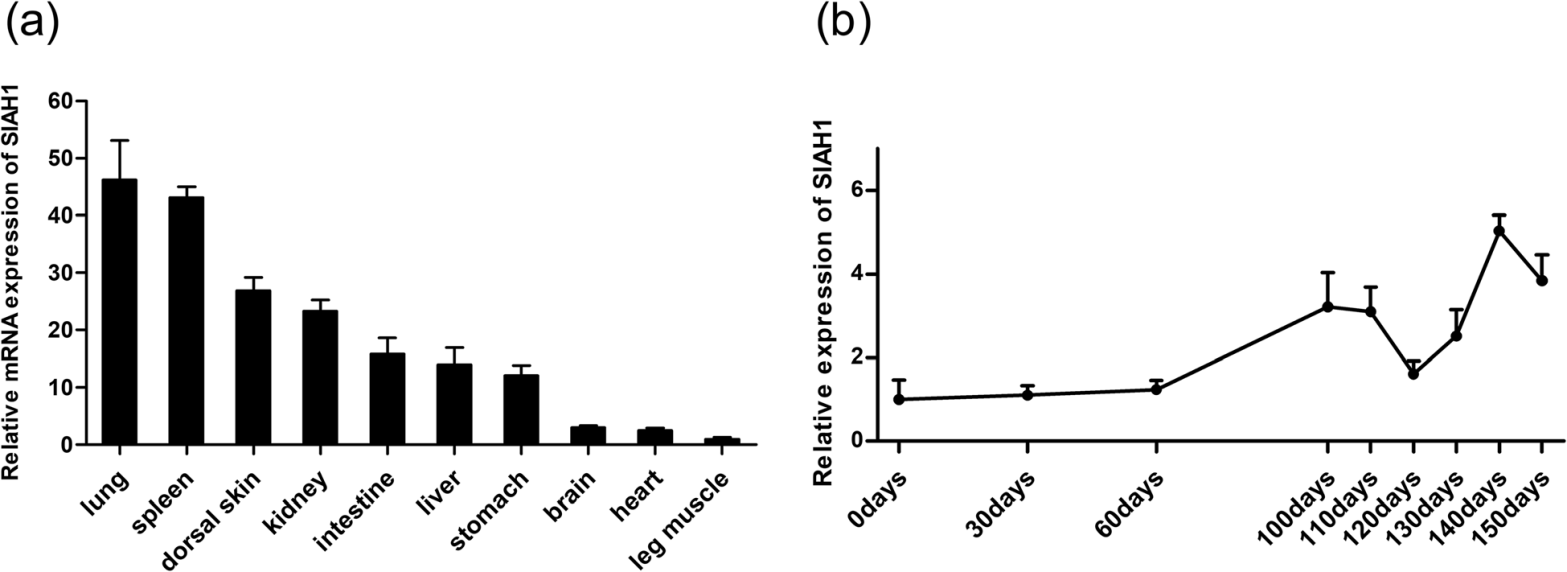
Relative expression levels of *SIAH1* in various tissues and hair follicle cycle. **a** Real-time RT-PCR was used to measure mRNA levels of *SIAH1* in different tissues. The data represent means ± SD (*n* = 9). Expression levels in the leg muscle were set to 1.0. **b** Relative expression levels of Angora rabbit *SIAH1* during hair cycling in the dorsal skin. Data represent means ± SD (n = 9). Expression level at day 0 was set to 1.0

Tissue distribution can provide useful insights into putative gene function. The expression of *SIAH1* mRNA was observed in all tissues examined with differential expression. The highest expression levels of *SIAH1* mRNA were detected in the lung, spleen, dorsal skin and kidney, with a moderate level of expression detected in the intestine, liver and stomach, while the least expression was observed in the brain, heart and leg muscles (Fig. 2a).

mRNA levels of *SIAH1* at were measured at 9 time points in the skin development and hair follicle cycle of the rabbit. These time points were selected based on the cycle of rabbit hair follicles, including anagen, catagen and telogen stages. Based on the rabbit hair follicle synchronization model, we found that the expression level of *SIAH1* in the regressive period of the rabbit hair cycle was significantly lower than that in the active growth and rest periods (Fig. 2b).

### Detection of *SIAH1* mRNA expression after its overexpression and interference

**Fig. 3:**
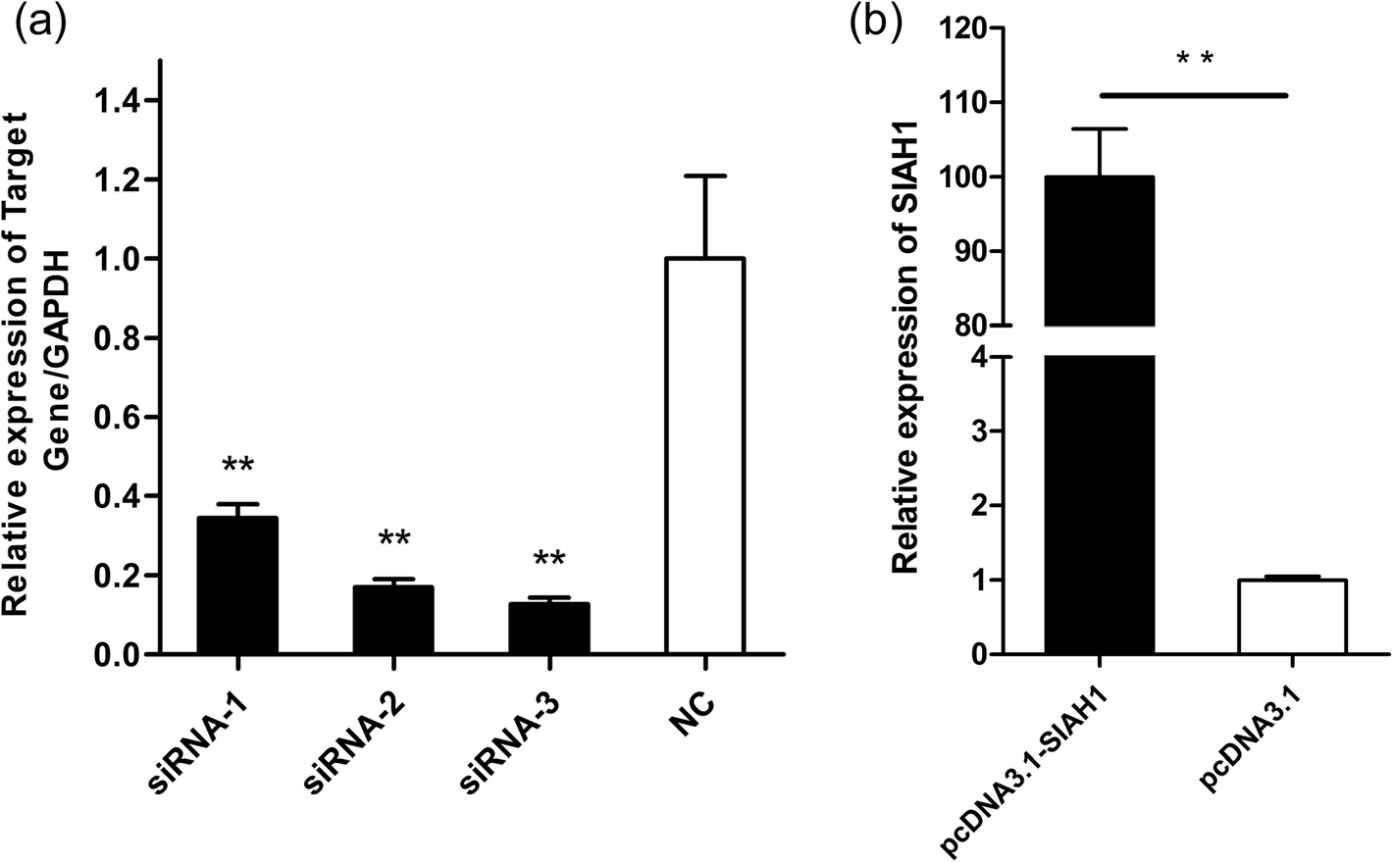
Expression levels of *SIAH1* mRNA in each group of cells. **a** The expression of siRNA-*SIAH1* in three groups was quantified. siRNA significantly reduced the expression of *SIAH1* in DP cells. **b** pcDNA3.1-*SIAH1* significantly reduced the expression of the *SIAH1* mRNA level in DP cells. ** indicates a statistically significant difference (*P* < 0.01)

In order to analyze the functions of *SIAH1*, three specific siRNAs were created and a pcDNA3.1-*SIAH1* overexpression vector established. By preparing nucleic acid-liposome complexes, pcDNA3.1-*SIAH1* and three siRNAs were transfected into DP cells and the expression of *SIAH1* quantified by qRT-PCR. Quantitative RT-PCR analysis using *GAPDH* as the endogenous control indicated that all three siRNAs reduced the expression of *SIAH1* mRNA but exhibited a different degree of interference compared with the siRNA-NC control group (Fig. 3a).

By comparing *SIAH1* mRNA levels in different siRNA groups, it was found that siRNA-3 had the greatest inhibitory effect on *SIAH1* mRNA expression levels, levels which were significantly lower than those of the siRNA-NC group, with inhibition ratios of 65.63, 82.98 and 87.23%, respectively (Table 1). In addition, we observed that the *SIAH1* mRNA expression levels in the pcDNA3.1-*SIAH1* group were significantly higher than those in the pcDNA3.1 group (Fig. 3b).

**Table 1:** Expression levels of *SIAH1* mRNA in different siRNA groups

Group	ΔCt	ΔΔCt	Relative expression	Inhibition Ratio (%)
siRNA-1	8.94 ± 0.15	1.52 ± 0.15	0.34^c^	65.63%
siRNA-2	9.95 ± 0.16	2.54 ± 0.16	0.17^b^	82.98%
siRNA-3	10.37 ± 0.17	2.96 ± 0.17	0.13^a^	87.23%
siRNA-NC	7.41 ± 0.26	0.00 ± 0.26	1.00^d^	–

Values with the same lower case letter indicate no significant difference (*P* > 0.05). Different letters indicate significant differences (*P* < 0.05). *CT* cycle threshold, *mRNA* messenger RNA, *siRNA* small interfering RNA

### Expression of skin and hair follicle development-related genes following overexpression and interference of *SIAH1*

**Fig. 4:**
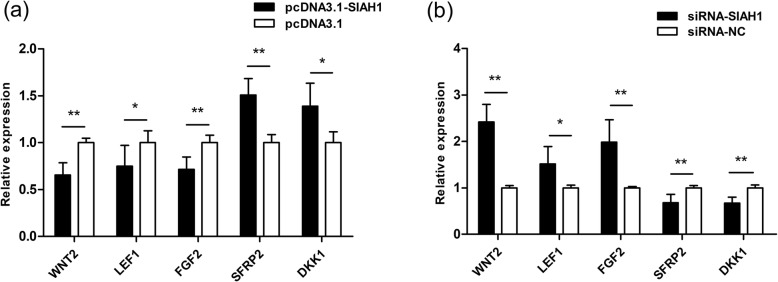
Effect of *SIAH1* expression on the expression levels of skin and hair follicle development-related genes. **a** Effect of pcDNA3.1-*SIAH1* on the expression levels of *WNT2*, *LEF1*, *FGF2*, *SFRP2* and *DKK1* genes. **b** Effect of siRNA-*SIAH1* on the expression levels of *WNT2*, *LEF1*, *FGF2*, *SFRP2* and *DKK1* genes. * indicates a statistically significant difference (*P* < 0.05), ** indicates a highly statistically significant difference (*P* < 0.01)

The pcDNA3.1-*SIAH1* vector and siRNA-2 with the highest inhibition ratio of *SIAH1* mRNA expression levels were transfected into DP cells, and the expression levels of skin and hair follicle development-related genes *WNT2*, *LEF1*, *FGF2*, *SFRP2* and *DKK1* quantified by qRT-PCR. The expression levels of these five genes changed significantly when *SIAH1* was overexpressed, with *WNT2*, *LEF1* and *FGF2* expression levels decreased and those of *SFRP2* and *DKK1* increased (*P* < 0.01) (Fig. 4a). Conversely, siRNA-*SIAH1* caused a significant increase in the expression levels of *WNT2*, *LEF1* and *FGF2*, with a significant decrease in expression levels of *SFRP2* and *DKK1* (*P* < 0.01) (Fig. 4b).

## Discussion

Genetic factors play a major role in determining traits in rabbit fur. Exploring the molecular mechanisms regulating skin and hair follicle development is currently a topic of considerable attention in medical and biological research [19, 20]. A previous study found that *SIAH1* was enriched in the Wnt signaling pathway and potentially related to hair follicle cycle and skin development [10]. Many studies have confirmed that *SIAH1* overexpression in cells decreases the expression of β-catenin through β-catenin degradation-dependent and independent mechanisms [14, 21, 22]. β-catenin is involved in the proliferation and differentiation of hair follicle stem cells and other biological regulation processes associated with skin and hair follicle development [23–25]. However, there is insufficient research evidence about the role of *SIAH1* in hair follicle cycling.

In the present study, we cloned and characterized the full-length of the cDNA sequence of *SIAH1* which was found to include a 188 bp 5′-UTR, an ORF of 948 bp and a 579 bp 3′-UTR. The predicted SIAH1 amino acid sequence was found to contain a conserved protein domain family, of the Seven in absentia (*Sina*) superfamily. Like its *Drosophila* Sina homologue, mammalian SIAH1 interacts with its target protein using the C-terminal substrate binding domain and labels it with ubiquitin. The protein complex then binds to the E2 protein via the N-terminal RING domain of SIAH1 leading finally to degradation of the substrate protein through the ubiquitin-proteasome degradation pathway [26–28]. Except for differences in length and sequence of the 32 AA N-terminal end of SIAH1 in different animals, the sequence in rabbits is highly conserved compared with other animals. The conserved sequence of SIAH1 contains two major functional domains, indicating that the role of SIAH1 may be stable across different species. Phylogenetic tree analysis again revealed that the sequence possessed close genetic relationships with these.

*SIAH1* mRNA was found to be widely expressed but at different levels in all tissues examined in our study. Wide differences in levels of *SIAH1* mRNA expression have also been observed in a range of adult and mouse tissues [29, 30]. Studies have found that *SIAH1* is also widely present in many cell lines and human tissues [31]. The extensive tissue distribution of *SIAH1* suggests that it may play an important cellular role. However, the high levels of *SIAH1* in dorsal skin suggests that it may have a genetic function in those sites. According to relevant research, the anagen, catagen and telogen phases of Angora rabbit hair after shaving occur at 0–110, 120–130 and 140–150 days, respectively [10]. Skin and hair follicle development-related genes play different roles at the various stages of hair follicle development. For example, *SFRP2* is principally expressed during the degenerative phase [8]. *DDK1* is an inhibitor of the classical Wnt signaling pathway and is mainly expressed during the growth phase [32]. *WNT2*, *LEF1* and *FGF2* are principally expressed during growth or degenerative phases [33–35]. In the present study, the mRNA expression levels of *SIAH1* were found to be significantly lower in the degenerative phase than in the growing and resting phases. Quantification of the expression of skin and hair follicle development-related genes indicated that there was a significant relationship between *SIAH1* and the other related genes. These may explain why *SIAH1* is mainly expressed in the growth and resting phases, and also suggests that *SIAH1* does not play an important role in the degenerative phase of hair follicles.

In order to explore whether *SIAH1* promotes or plays an inhibitory role in the hair cycling of the rabbit, several skin and hair follicle development-related genes were selected for functional verification. *WNT2* plays an important role in regulating hair length in hair follicle morphogenesis and may be important for hair follicle initiation [33, 36]. *LEF1* mediates cell division and migration by mediating Wnt signaling by binding to β-catenin [34]. *FGF2* promotes the proliferation of dermal papillae and mitosis of true skin fibroblasts [35]. During catagen of hair follicles, *SFRP2* affects skin and hair follicle development by inhibition of keratinocyte proliferation and Wnt activity [8] and *DKK1* has been shown to promote the regression of hair follicles [37]. The expression levels of all these genes changed significantly after overexpression of *SIAH1*. However, this change showed two trends: the expression levels of *WNT2*, *LEF1* and *FGF2* decreased, but those of *SFRP2* and *DKK1* increased. After inhibition of *SIAH1* expression, the expression levels of those genes exhibited a change that was opposite. Therefore, we believe that *SIAH1* inhibits the skin development and hair follicle cycle.

## Conclusions

In conclusion, we successfully amplified the complete *SIAH1* sequence based on the existing predicted sequences and analyzed the structure, identity and phylogeny of the amino acid sequence. The expression of *SIAH1* in hair cycling and in different tissues was examined, revealing that *SIAH1* is highly expressed in the lung, spleen, dorsal skin and kidneys, with expression levels of *SIAH1* in the regressive period significantly lower than that in the active growth and rest periods. Measurement of the expression levels of other skin and hair follicle development-related genes after overexpression and interference of *SIAH1* mRNA revealed that the gene can affect skin and hair follicle development and exert an inhibitory effect. These results could provide foundamental insights into the role of *SIAH1* as a target gene in rabbit skin and hair follicle development.

## Materials and methods

### Animals and sample collection

The Anhui Academy of Agricultural Sciences provided 6-month-old male Wanxi Angora rabbits. All animal procedures were approved by the Animal Care and Use Committee of Yangzhou University. The hair on the back of each rabbit was shaved using an electric shaver until the light pink skin was exposed, causing the structure of the hair follicles to be destroyed and resulting in them starting a complete hair follicle cycle. The experimental Angora rabbits were housed in an appropriate, clean and disease-free environment and had free access to water and pellet feed. The temperature and relative humidity were regulated to 15–25 °C and 40–60%, respectively. Rabbit health was monitored and recorded twice daily (7:00 and 18:00). Rabbits were sacrificed with intravenous injection of air. One cm^2^ of skin on the back of each experimental rabbit was harvested into a sample tube and placed quickly in liquid nitrogen, at days 0, 30, 60, 100, 110, 120, 130, 140 and 150. In addition, samples of the heart, liver, spleen, lung, kidney, brain, stomach, small intestine, dorsal skin and leg muscle were collected from three healthy rabbits at 180 days of age. Similarly, these samples of tissue were quickly placed in liquid nitrogen to prevent RNA degradation, then stored in a − 80 °C freezer for total RNA extraction.

### Full-length acquisition of *SIAH1*

The full length of the *SIAH1* gene was obtained using three 5′-RACE primers (GSP1, GSP2 and GSP3) and two 3′-RACE primers (C396–1 and C396–2) that were designed by reference to the amplified CDS sequence. The 5′ and 3′ end sequences were amplified using a SMARTer™ RACE cDNA Amplification kit (Clontech, China) in accordance with the manufacturer’s instructions. The amplified product was subjected to electrophoresis and the target band purified. After purification, the product was ligated to a pMD-19 T vector, then the positive clone selected for sequencing. Sequences of the primers are displayed in Table 2.

**Table 2:** Primers used for RACE

Name	Sequences of primers (5′-3′)	Application
GSP1	TCTGCTGAAGAACTGC	5′-RACE
GSP2	TCAGGGGCAATCCTCCAC	5′-RACE
GSP3	GAGAGTGTACTAGGAGTCAAG	5′-RACE
C396–1	ATGGGCACAGGCGACGATTGACTT	3′-RACE
C396–2	GAGGGAATTGCGACAGCCATCATG	3′-RACE

### Sequence analysis of *SIAH1*

Sequence analysis of the Angora rabbit *SIAH1* was performed by ExPaSy software (https://web.expasy.org/protparam/) [38]. Protein sequence alignments were performed using Clustal (https://www.ch.embnet.org/) [39]. A phylogenetic tree was constructed using MEGA 7.0 software [40] Kumar using the neighbor-joining method. Structural domains were predicted using SMART (http://smart.embl-heidelberg.de/) [41] and the secondary structure of the protein predicted using the online software SOPMA (https://prabi.ibcp.fr/htm/site/web/) [42]. Protein signal peptides were predicted using SignalP 4.1 (http://www.cbs.dtu.dk/services/SignalP-4.1/) [43]. Protein transmembrane domains were predicted by TMHMM (http://www.cbs.dtu.dk/services/TMHMM/) [44]. Predictive analysis of subcellular localization of the *SIAH1* was conducted using PSORT II Prediction (https://psort.hgc.jp/form2.html) [45].

### Cell culture and transfection

Dermal papilla cell lines that were established by our research group were used to consuct cell experiments. DP cells were cultured in mesenchymal stem cell medium (ScienCell®) containing 5% fetal bovine serum, 1% mesenchymal stem cell growth supplement and 1% penicillin/streptomycin solution. The cells were cultured in an incubator at 37 °C within an atmosphere containing 5% CO_2_ and a high relative saturation humidity (95%). The DP cells were seeded into 24-well cell culture plates then transfected with Lipofectamine™ 3000 (Invitrogen, CA) when 80% confluent, in accordance with the manufacturer’s recommended protocol.

### Quantitative real-time polymerase chain reaction

Total RNA was extracted from the cultured cells and skin and tissue samples using an RNA extraction kit (Tiangen, China). Approximately 1 μg of total RNA was synthesized into complete cDNA for qRT-PCR using HiScript reverse transcriptase (Vazyme, China). The reverse transcription reaction contained 1 μg of total RNA and 4 μL of 4 × gDNA wiper mix supplemented with ddH_2_O to 16 μL. The first step reaction mixture was incubated at 42 °C for 2 min, then 4 μL of 5 × qRT SuperMix were added to the 16 μL mixture which was incubated at 50 °C for 15 min then 85 °C for 3 min to synthesize cDNA. The template DNA was analyzed using qRT-PCR with ChamQ™ SYBR® qPCR Master Mix (Vazyme). The 20 μL PCR reaction included 10.0 μL 2 × ChamQ SYBR qPCR Master Mix, 0.4 μL of each specific forward and reverse primer (10 μM), 0.4 μL of 50 × ROX Reference Dye 2, 1.0 μL of cDNA and 7.8 μL of ddH_2_O. The data were processed using a QuantStudio® 5 real-time PCR system (Applied Biosystems). The 2^-ΔΔCt^ method [46] was used to calculate the relative expression levels of genes, the resulting data normalized against the endogenous control glyceraldehyde-3-phosphate dehydrogenase (*GAPDH*). All primers were designed using Primer Premier 5.0 software and synthesized by Tsingke Biological Company (Beijing, China) (Table 3).

**Table 3:** Primer sequences for qRT-PCR

Name	Sequences of primers (5′-3′)
*GAPDH*	F: CACCAGGGCTGCTTTTAACTCT R: CTTCCCGTTCTCAGCCTTGACC
*SIAH1*	F: ACGACCGCGTCCAACAATGA R: AGCTTTGGGCGACAGTTGCT
WNT2	F: AGCCATCCAGGTCGTCATGAACCAG R: TGCACACACGACCTGCTGTACCC
LEF1	F: CATCTCGGGTGGATTCAGG R: ATGAGGGATGCCAGTTGTG
FGF2	F: GTGTGTGCAAACCGTTACCTT R: TCGTTTCAGTGCCACATACCAG
SFRP2	F: CCAGCCCGACTTCTCCTACAAGC R: TCCAGCACCTCTTTCATGGTCT
DKK1	F: CACAGAGGACGAGGAGTGTG R: CTTCCTGCAAGCCAGACAGA

*F* forward primer, *R* reverse primer

### Overexpression and interference of *SIAH1*

First strand cDNA was synthesized from the total RNA from 1 μg of high-quality rabbit skin using a HiScript® II1st Strand cDNA Synthesis Kit (Vazyme). Primers were designed from the rabbit *SIAH1* CDS sequence (GenBank accession no. XM_008275061.2). The 50 μL PCR reaction mixture contained 1 μL cDNA, 2 μL forward primer (10 μM), 2 μL reverse primer (10 μM), 1 μL Phanta Max Super-Fidelity DNA Polymerase, 1 μL dNTP Mix, 25 μL Phanta Max Buffer and 18 μL ddH_2_O. The PCR product was identified using a 1.5% agarose gel and the specific fragment recovered and purified using an agarose gel DNA extraction kit (TaKaRa, China). The *SIAH1* CDS sequence was subcloned into *Hind*III and *EcoR*I digested pcDNA 3.1 vector (Invitrogen), which was termed pcDNA3.1-*SIAH1*. The siRNA-*SIAH1* and siRNA-NC were purchased from GenePharma (Shanghai, China). The siRNA sequence is detailed in Table 4.

**Table 4:** *SIAH1* overexpression and interference related primer sequences

Name	Sequences of primers (5′-3′)
*SIAH1* CDS	F: CCC*AAGCTT*ATGATAGAAAGAAACTTGACTCCTA R: CCG*GAATTC*TCAACACATGGAAATCGTTACATTG
siRNA-1	F:GCACGACCGCGUCCAACAATT R:UUGUUGGACGCGGUCGUGCTT
siRNA-2	F:CCUCGUCUGGAUGUGAAAUTT R:AUUUCACAUCCAGACGAGGTT
siRNA-3	F:CCAGCAGUUCUUUGCAAUUTT R:AAUUGCAAAGAACUGCUGGTT
siRNA-NC	F:UUCUCCGAACGUGUCACGUTT R:ACGUGACACGUUCGGAGAATT

Bases in italics and underlined represent the enzyme cutting site. *F* forward primer, *R* reverse primer

### Statistical analysis

Statistical analysis was performed using SPSS v21.0 software (IBM Corporation, Armonk, USA). All data were presented as means ± SD. Depending on the various experimental requirements, either a one-way analysis of variance or t-test was conducted to analyze the statistical significance of the experimental data, with *P* < 0.05 considered the criterion for statistical significance.

## Data Availability

The authors declare that the data supporting the findings of this study are available within the articles.

## Abbreviations

SIAH1: Seven in absentia homolog 1
mRNA: messenger RNA
siRNA: small interfering RNA
qRT-PCR: quantitative real-time polymerase chain reaction
RACE: Rapid amplification of cDNA ends
DP: Dermal papilla
CT: Cycle threshold
P: *P* value
SD: Standard deviation

## References

[1] Hardy MH (1992). The secret life of the hair follicle. Trends Genet.

[2] Paus R, Cotsarelis G (1999). The biology of hair follicles. N Engl J Med.

[3] Oshima H, Rochat A, Kedzia C, Kobayashi K, Barrandon Y (2001). Morphogenesis and renewal of hair follicles from adult multipotent stem cells. Cell..

[4] Jeong KH, Joo HJ, Kim JE, Park YM, Kang H (2015). Effect of mycophenolic acid on proliferation of dermal papilla cells and induction of anagen hair follicles. Clin Exp Dermatol.

[5] Morgan BA (2014). The dermal papilla: an instructive niche for epithelial stem and progenitor cells in development and regeneration of the hair follicle. Cold Spring Harb Perspect Med.

[6] Adly MA, Assaf HA, Hussein MR (2006). Expression of the heat shock protein-27 in the adult human scalp skin and hair follicle: hair cycle-dependent changes. J Am Acad Dermatol.

[7] Higgins CA, Petukhova L, Harel S, Ho YY, Drill E, Shapiro L, Wajid M, Christiano AM (2014). FGF5 is a crucial regulator of hair length in humans. Proc Natl Acad Sci U S A.

[8] Kim BK, Yoon SK (2014). Expression of sfrp2 is increased in catagen of hair follicles and inhibits keratinocyte proliferation. Ann Dermatol.

[9] Qi Y, Li M, Xu L, Chang Z, Shu X, Zhou L (2016). Therapeutic role of human hepatocyte growth factor (HGF) in treating hair loss. PeerJ..

[10] Zhao B, Chen Y, Hu S, Yang N, Wang M, Liu M, Li J, Xiao Y, Wu X (2019). Systematic analysis of non-coding RNAs involved in the angora rabbit (Oryctolagus cuniculus) hair follicle cycle by RNA sequencing. Front Genet.

[11] Hu G, Zhang S, Vidal M, Baer JL, Xu T, Fearon ER (1997). Mammalian homologs of seven in absentia regulate DCC via the ubiquitin-proteasome pathway. Genes Dev.

[12] Lin C, Yuan Y, Chen X, Li H, Cai B, Liu Y, Zhang H, Li Y, Huang K (2015). Expression of Wnt/β-catenin signaling, stem-cell markers and proliferating cell markers in rat whisker hair follicles. J Mol Histol.

[13] Matsuzawa SI, Reed JC (2001). Siah-1, SIP, and Ebi collaborate in a novel pathway for beta-catenin degradation linked to p53 responses. Mol Cell.

[14] Liu J, Stevens J, Rote CA, Yost HJ, Hu Y, Neufeld KL, White RL, Matsunami N (2001). Siah-1 mediates a novel beta-catenin degradation pathway linking p53 to the adenomatous polyposis coli protein. Mol Cell.

[15] Maeda A, Yoshida T, Kusuzaki K, Sakai T (2002). The characterization of the human Siah-1 promoter. FEBS Lett.

[16] Hara MR, Snyder SH (2006). Nitric oxide-GAPDH-Siah: a novel cell death cascade. Cell Mol Neurobiol.

[17] Jumpertz S, Hennes T, Asare Y, Vervoorts J, Bernhagen J, Schutz AK (2014). The beta-catenin E3 ubiquitin ligase SIAH-1 is regulated by CSN5/JAB1 in CRC cells. Cell Signal.

[18] Li Q, Wang P, Ye K, Cai H (2015). Central role of SIAH inhibition in DCC-dependent cardioprotection provoked by netrin-1/NO. Proc Natl Acad Sci U S A.

[19] Sardella C, Winkler C, Quignodon L, Hardman JA, Toffoli B, Giordano Attianese GMP, Hundt JE, Michalik L, Vinson CR, Paus R, Desvergne B, Gilardi F (2018). Delayed hair follicle morphogenesis and hair follicle dystrophy in a Lipoatrophy mouse model of Pparg Total deletion. J Invest Dermatol.

[20] Shirokova V, Biggs LC, Jussila M, Ohyama T, Groves AK, Mikkola ML (2016). Foxi3 deficiency compromises hair follicle stem cell specification and activation. Stem Cells.

[21] Yoshibayashi H, Okabe H, Satoh S, Hida K, Kawashima K, Hamasu S, Nomura A, Hasegawa S, Ikai I, Sakai Y (2007). SIAH1 causes growth arrest and apoptosis in hepatoma cells through beta-catenin degradation-dependent and -independent mechanisms. Oncol Rep.

[22] Dimitrova YN, Li J, Lee YT, Eateves JR, Freidman DB, Choi HJ, Weis WI, Wang CY, Chazin WJ (2010). Direct ubiquitination of beta-catenin by Siah-1 and regulation by the exchange factor TBL1. J Biol Chem.

[23] Huelsken J, Vogel R, Erdmann B, Cotsarelis G, Birchmeier W (2001). Beta-catenin controls hair follicle morphogenesis and stem cell differentiation in the skin. Cell..

[24] Choi YS, Zhang Y, Xu M, Yang Y, Ito M, Peng T, Zheng C, Nagy A, Hadjantonakis AK, Lang RA, Cotsarelis G, Andl T, Morrisey EE, Millar SE (2013). Distinct functions for Wnt/beta-catenin in hair follicle stem cell proliferation and survival and interfollicular epidermal homeostasis. Cell Stem Cell.

[25] Lo Celso C, Prowse DM, Watt FM (2004). Transient activation of beta-catenin signalling in adult mouse epidermis is sufficient to induce new hair follicles but continuous activation is required to maintain hair follicle tumours. Development..

[26] Hu G, Fearon ER (1999). Siah-1 N-terminal RING domain is required for proteolysis function, and C-terminal sequences regulate oligomerization and binding to target proteins. Mol Cell Biol.

[27] Frew IJ, Huang HL, Wiche G, Traficante N, Nice E, Catimel B, Bowtell DD, House CM (2003). A binding motif for Siah ubiquitin ligase. Proc Natl Acad Sci U S A.

[28] Santelli E, Leone M, Li C, Fukushima T, Preece NE, Olson AJ, Ely KR, Reed JC, Pellecchia M, Liddington RC, Matsuzawa S (2005). Structural analysis of Siah1-Siah-interacting protein interactions and insights into the assembly of an E3 ligase multiprotein complex. J Biol Chem.

[29] Hu G, Chung YL, Glover T, Valentine V, Look AT, Rearon ER (1997). Characterization of human homologs of the Drosophila seven in absentia (sina) gene. Genomics..

[30] Della NG, Senior PV, Bowtell DD (1993). Isolation and characterisation of murine homologues of the Drosophila seven in absentia gene (sina). Development..

[31] Bruzzoni-Giovanelli H, Fernandez P, Veiga L, Podgorniak MP, Powell DJ, Candeias MM, Mourah S, Calvo F, Marín M (2010). Distinct expression patterns of the E3 ligase SIAH-1 and its partner kid/KIF22 in normal tissues and in the breast tumoral processes. J Exp Clin Cancer Res.

[32] Andl T, Reddy S, Gaddapara T, Millar SE (2002). Wnt signals are required for the initiation of hair follicle development. Dev Cell.

[33] Nie Y, Li S, Zheng X, Chen W, Li X, Liu Z, Hu Y, Qiao H, Qi Q, Pei Q, Cai D, Yu M, Mou C (2018). Transcriptome reveals long non-coding RNAs and mRNAs involved in primary wool follicle induction in carpet sheep fetal skin. Front Physiol.

[34] Nusse R (2005). Wnt signaling in disease and in development. Cell Res.

[35] Kiso M, Hamazaki TS, Itoh M, Kikuchi S, Nakagawa H, Okochi H (2015). Synergistic effect of PDGF and FGF2 for cell proliferation and hair inductive activity in murine vibrissal dermal papilla in vitro. J Dermatol Sci.

[36] Yue Y, Guo T, Yuan C, Liu J, Guo J, Feng R, Niu C, Sun X, Yang B (2016). Integrated analysis of the roles of long noncoding RNA and coding RNA expression in sheep (Ovis aries) skin during initiation of secondary hair follicle. PLoS One.

[37] Kwack MH, Kim MK, Kim JC, Sung YK (2012). Dickkopf 1 promotes regression of hair follicles. J Invest Dermatol..

[38] Artimo P, Jonnalagedda M, Arnold K, Baratin D, Csardi G, de Castro E, Duvaud S, Flegel V, Fortier A, Gasteiger E, Grosdidier A, Hernandez C, Ioannidis V, Kuznetsov D, Liechti R, Moretti S, Mostaguir K, Redaschi N, Rossier G, Xenarios I, Stockinger H (2012). ExPASy: SIB bioinformatics resource portal. Nucleic Acids Res.

[39] Larkin MA, Blackshields G, Brown NP, Chenna R, McGettigan PA, McWilliam H, Valentin F, Wallace IM, Wilm A, Lopez R, Thompson JD, Gibson TJ, Higgins DG (2007). Clustal W and Clustal X version 2.0. Bioinformatics..

[40] Kumar S, Stecher G, Tamura K (2016). MEGA7: molecular evolutionary genetics analysis version 7.0 for bigger datasets. Mol Biol Evol.

[41] Letunic I, Bork P (2018). 20 years of the SMART protein domain annotation resource. Nucleic Acids Res.

[42] Geourjon C, Deleage G (1995). SOPMA: significant improvements in protein secondary structure prediction by consensus prediction from multiple alignments. Comput Appl Biosci.

[43] Petersen TN, Brunak S, von Heijne G, Nielsen H (2011). SignalP 4.0: discriminating signal peptides from transmembrane regions. Nat Methods.

[44] Tatusov RL, Koonin EV, Lipman DJ (1997). A genomic perspective on protein families. Science..

[45] Nakai K, Horton P (1999). PSORT: a program for detecting sorting signals in proteins and predicting their subcellular localization. Trends Biochem Sci.

[46] Schmittgen TD, Livak KJ (2008). Analyzing real-time PCR data by the comparative CT method. Nat Protoc.

